# Prevalence of intestinal parasitic infections and associated factors among street children in Jimma town; south West Ethiopia in 2019: a cross sectional study

**DOI:** 10.1186/s12889-019-8083-4

**Published:** 2019-12-23

**Authors:** Sabit Zenu, Eshetu Alemayehu, Kifle Woldemichael

**Affiliations:** 1 Department of Public Health, Mettu University, Metu, Ethiopia; 2 Department of Epidemiology, Jimma University, Jimma, Ethiopia

**Keywords:** Street children, Intestinal parasites, Jimma town

## Abstract

**Background:**

Street child is any child whose age is less than 18 years for whom the street has become his or her habitual abode and/or source of livelihood, is inadequately protected, supervised or directed by responsible adults. In Ethiopia the health problems of street children are given poor attention in research. This problem is pronounced when it comes to intestinal parasitic infections, making it difficult to design appropriate interventions targeting this segment of population. The aim of this study was to assess the prevalence of intestinal parasitic infections and associated factors among street children in Jimma town in the year 2019.

**Methods:**

Community based cross sectional study was employed. Complete enumeration was used to include 312 street children. Pretested questionnaire was used to collect the data. Data was entered to Epidata version 3.1 and exported to SPSS version 20. Stool samples were examined by wet mount and formalin ether concentration techniques. Bivariable and multivariable logistic regression was used to identify factors associated with intestinal parasitic infection. Significance of association was decided by using the 95% confidence interval of AOR and *P*-value of ≤0.05 in the multivariable model.

**Result:**

A total of 312 children of the street were involved in the study making the response rate 96.2%. The prevalence of intestinal parasitic infection was 66.7%. Untrimmed finger nails AOR = 2.03;95%CI (1.02–4.06), eating street food AOR = 2.24;95% CI (1.04–5.02), practice of swimming in unprotected water bodies AOR = 2.5; 95% CI (1.24–5.04), not wearing shoes at the time of data collection AOR = 3.8;95% CI (1.8–8.2) and lacking knowledge of way of transmission of intestinal parasites AOR = 2.5; 95% CI (1.25–5.0) were significantly associated with parasitic infections.

**Conclusions:**

The prevalence of intestinal parasitic infections among street children in the study area was high and require integrated interventions to avert the problem. Several factors were also found to be associated with intestinal parasitic infections. Measures has to be taken to curb the problem by including them in mass drug administration and targeted health education towards identified factors.

## Background

Intestinal parasitosis refers to a group of diseases caused by one or more species of protozoa, cestodes, trematodes and nematodes. These parasites are responsible for the major share of morbidity and mortality in communities where there is over-crowding, poor environmental sanitation and personal hygienic practices, which make them a great concern for the developing countries. The greatest brunt of the disease is shared by children aged 12–14 years [1]. Factors ranging from water, sanitation and hygiene to weak health service delivery to populations at risk of infections were major reasons for persistence of these diseases in the community [2].

Intestinal parasitic infections (IPIs) are one of the most common diseases and resulted in widespread morbidity starting from early times of human history. The eggs of certain intestinal worms were recovered from mummified feces of humans dating back thousands of years [3]. These days intestinal parasites, mostly of soil transmitted helminthiasis, affect nearly a third of global population and severely affects underprivileged populations of developing countries where poverty, undernutrition, inadequate sanitation and lack of clean drinking water prevails [1, 4]. In 2010, an estimated 438.9 million people were infected with Hookworms, 819.0 million with *Ascaris lumbricoides*, 464.6 million with *Trichuris trichuria* and these parasites inflicted nearly 5 million years lived with disability in the world [5].

The effect of this group of disease is mainly manifested as a chronic and insidious effect on health and quality of life, while intense infection can result in developmental faltering, poor growth and poor school performance [1]. Studies indicate that children with heavy intestinal parasitic infection have lower body mass index, lower hemoglobin levels and are often stunted [6]. Some studies also revealed that children with heavy parasitic infection to have poor anthropometric indices, growth retardation, poor cognitive development, chronic inflammatory diseases and life threatening surgical conditions. Furthermore, the chemotherapeutic treatment of the intestinal parasites resulted in improved physical, motor and language development [7–9].

The effect of intestinal parasitic infection on health of infected children is found to depend on the status of the children. Children with underlying nutritional problems and other health conditions are more likely to suffer from the brunt of the diseases and develop morbidities [10].

According to WHO, three quarters of children with intestinal worms requiring chemotherapy are in South East Asia and African regions. In Africa only, nearly three hundred million preschool age and school age children require preventive chemotherapy; this figure accounts for 30% of the global chemotherapy requirement [1].

In Ethiopia the prevalence of intestinal worms among school aged children ranged from 20 to 100% with the highest percentage in wet and humid central areas while the prevalence is found to be limited in arid and dry areas of the country. In Ethiopia intestinal parasites are endemic in 329 districts with about 6545 Kebeles and 11,410 schools [11].

Street children are defined by the United Nations as “boys and girls for whom ‘the street’ (including unoccupied dwellings, wasteland, etc.) has become their home and/or source of livelihood, and who are inadequately protected or supervised by responsible adults.” Additionally, the organization categorizes “street children” as either children on the street, who worked on the street and went home to their families at night and children of the street, who lived on the street, were functionally without family support who lived completely on their own [12].

There are wide controversies concerning the reliable estimate of the number of the street children around the world. The widely contested claim of the United Nations International Children’s Fund (UNICEF) stating the figure at 100 million is now rendered baseless and currently the estimate is stated in the area of tens of millions with rapidly increasing pattern due to a rapidly urbanizing and growing global population. Together with increasing inequalities and migration, studies suggest that numbers are generally increasing, including in richer regions. Studies suggest factors like war, HIV/AIDS, economic and social disintegration, family separation and abuse for increasing pattern of the number of street children [12, 13].

The precise estimate of number of street children in Ethiopia is also controversial. In 2007 the ministry of Labor and Social Affairs conducted a study that is supported by the UNICEF and estimated the overall number of children on or off the street at around 150,000 with about 60,000 living in the capital. The recent estimates of the number of street children as 500,000–700,000 is roughly five times higher than the report in 2007 and approaches two to three times the population of Jimma town. Efforts to curb the increasing number of street children in Ethiopia were largely ineffective due to fragmented interventions, increasing effect of push and pull factors on children and rapid urbanization of the country [14].

Several studies indicated that street children are disproportionately affected with range of diseases and health problems including parasitic infections, infestations, other infectious diseases, unintentional injuries, violence, abuse by older adults and police officers, substance abuse, malnutrition, sexually transmitted diseases, other reproductive and mental health problems. In all categories of infection fulltime street residents are far greatly affected than those on the street children [15–18].

Despite the wide-ranging health problems of the street children, only few studies are conducted to quantify the situation in Ethiopia on this segment of population and especially limited when it comes to the issue of intestinal parasitic infections. Most studies conducted to assess the prevalence of intestinal parasites in Ethiopia are conducted in institutions like schools. Some studies that are conducted to assess the prevalence of these diseases in Ethiopia are also carried out among the overall street residents and some on street beggars and not specifically on street children. This shows the paucity of information on the prevalence and factors associated with intestinal parasitic infections on street children, a group of children that are most likely to suffer from these conditions than adults and even than supervised children.

Accordingly, the objective of this study was to assess the prevalence and factors associated with intestinal parasitic infections among street children to bridge the knowledge gap and provide the rationale to reconsider the ongoing intervention modalities in the way that this segment of population will also be included.

## Methods

### Study area, design and period

The study was conducted in Jimma town by employing community based cross sectional study design from March 1–31/ 2019. Jimma town, capital of Jimma zone is located at 356 Km to South West of the national capital, Addis Ababa. Its astronomical location is 7° 4′ North Latitude and 36° 5′ East Longitude. In the year 2018, the total population of Jimma town is projected to be 205,384. From these 104,745 are females while 100,639 were males. Regarding the health service organizations, there are ten public health facilities in the town; among them are two hospitals and four health centers. Few organizations work to provide essential services to the street children in Jimma town. The biggest and most organized one is Faya Integrated Development Organization (FIDO) which provides comprehensive services ranging from legal aid to medical treatment. The organization has decentralized establishment including thirteen community social workers each at every urban Kebeles and registered community volunteers ranging 20–30 on each Kebeles. These community volunteers work under close supervision from community social workers and have direct contact with the street children.

### Sample size determination and sampling technique

Sample size was calculated for the two objectives separately and the highest sample size was used. Sample size for the first objective was calculated by using single population proportion formula by using 95% confidence interval, 5% margin of error and the prevalence of intestinal parasitic infections among street residents in Addis Ababa as 71.8%(19).

Accordingly, = **z**(**α**/**2**)^**2**^∗***P***(**1**−***P***)/ **D**^**2**^=(**1.96**)^**2**^∗0.718(**1**−**0**.718)/**0.05**^2^=311

Sample size for the second objective was calculated by using STATCALC of Epi info 7 for different variables and the highest among these resulted in sample size of 310 participants. Sample size of the first objective was higher (311). This sample size was chosen and corrected for finite population.

Since the source population is 365 from the preliminary survey which is less than 10,000, correction formula was used to adjust the sample size. Adding 10% for non-response rate, the final sample size was 185.

Complete enumeration was used after developing list of children of the street and included all 312 children who fulfilled the inclusion criteria. Thirteen urban Kebeles of Jimma town were included in the preliminary survey. By using Community social workers and volunteers at every Kebele, list of street children was developed for every Kebele. Demographic information, time since started living on the street, area of usual residence, means of communication, language, visible deformities, nick name and whether they go to home at the night or not was recorded on the format. Demographic information was also written on respondent card, provided to the respondent and told to come with for data collection.

### Data collection procedures and laboratory examinations

Demographic and other personal data were collected by interviewer administered questions using a structured questionnaire that was developed after reviewing relevant literatures. Selected children were brought to the Higher II Health Center by community volunteers. Questionnaire based questions were asked and recorded in shady and calm areas of the health center. Fresh stool sample was collected from every selected street child by laboratory technologists at the health center up on arrival. Small portion of the fresh stool sample was examined by using wet mount and the rest was fixed and examined by using formalin ether concentration method to determine the presence of parasitic eggs, trophozoites, cysts or relevant developmental stages of intestinal parasites.

Four licensed clinical nurses were employed to collect interview- based information from selected study participants and a BSc health officer supervised their activities. Laboratory examinations were conducted by two licensed laboratory technologist in Higher II Health Center medical laboratory.

### Data analysis procedures

Data was entered in to Epidata version 3.1 and exported to the SPSS version 20 for analysis. Data exploration was conducted to assess the completeness, and descriptive statistics were used to describe the data depending on its nature. The data were displayed by using tables and graphs. Inferential statistical analysis was conducted by using the bi variable and multivariable logistic regression. Variables with the level of significance ≤0.25 on the bi variable analysis were candidates for the multivariable analysis. Significance of the association was decided by using the *P*-value of ≤0.05 at the 95% confidence interval for the multivariable model.

### Data quality management

Licensed clinical nurses were selected for the collection of the interview-based data. The questionnaire prepared in English was translated into Amharic and translated back to English to assess consistency. The Amharic version was used while carrying out the interview. Intensive training was provided for the data collectors concerning the objectives of the study and the nature of study participants for 2 days. On site supervision of the data collectors and data collection process were carried out on daily basis. Data were checked for completeness and consistency, edited and coded on daily basis. Finally, it was cleaned after entry into computer and exported to SPSS version 20 for analysis.

Stool samples were collected by laboratory technologists, coded and examined without any delay. Quality control of laboratory examinations were carried out by involving two laboratory technologists at the health center. Concordant findings are taken as final measure and discordant findings were re-examined by a senior laboratory technologist and finally his conclusion was used as a final decision on the specimen. Questionnaires and stool specimens were coded and later re-merged with laboratory findings.

### Ethical consideration

Ethical clearance was sought from institutional review board of Jimma University; Institute of Health. Letter of cooperation was written to administration of the town. Permission was secured from the town’s Police department, and Office of Women, Children and Youth affairs to study street children. Assent was obtained from children aged 12–18 years; informed consent was sought for those aged 18 years after providing comprehensive information about the nature, objectives, risks and benefits of the study. Standard treatment was provided for children in which the intestinal parasites are diagnosed in addition to the provision of deworming for entire participants. Health information was disseminated to participants after the completion of the data collection.

## Result

### Sociodemographic and other characteristics of street children

**Fig. 1:**
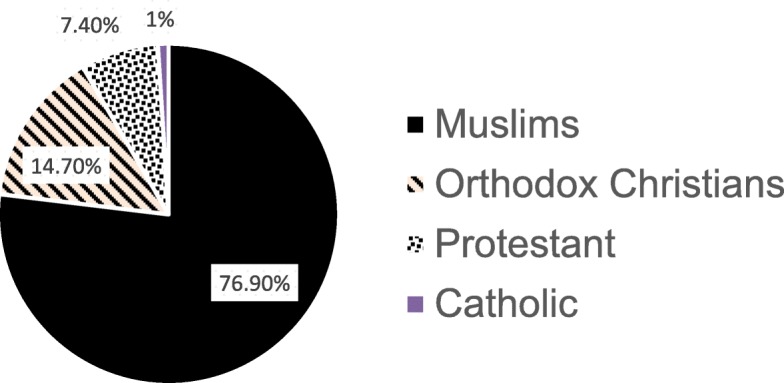
Religion of street children included in the study in Jimma town, 2019

A total of 324 children of the street fulfilled the inclusion criteria. Out of these, 12 were lost from back tracing and the study included 312 street children making the response rate 96.2%. Most of the studied children were male with a total of 284(91%). The median age of participants was 14 years with interquartile range (IQR) of 2 years (13–15 years). Most of children were ethnic Oromo 242(77.6%) and Muslims in religion 240(76.9%) (Fig. 1). Median duration of time since joining the street life was 12 months with (IQR) of 17.75 months (6.25–24 months). More than a two third of the children came from rural areas (Table 1).

**Table 1:** Socio demographic characteristics of street children included in the study in Jimma town, 2019

Characteristics	Categories		Frequencies (*n* = 312)	Percentages	Remark
Sex	Male		284	91%	
	Female		28	9%	
	Total		312	100%	
Ethnicity	Oromo		242	77.6%	
	Amhara		17	5.4%	
	Dawro		30	9.6%	
	Keficho		18	5.8%	
	Guraghe		4	1.6%	
	Tigre		1	0.3%	
	Total		312	100%	
Birth area	Urban		83	26.6%	
	Rural		229	73.4%	
	Total		312	100%	
Educational status	Never joined		79	25.3%	
	Able to read and write		23	7.4%	
	Grade 1–4		155	49.7%	
	Grade 5–8		55	17.6%	
	Total		312	100%	
Current school attendance	Yes		19	6.1%	
	No		293	93.9	
	Total		312	100%	
Working on the street	Yes		309	99%	
	No		3	1%	
	Total		312	100%	
Type of income generating works	Carrying goods	Yes	226	72.4%	
		No	83	26.6%	
		Total	309	100%	
	Begging	Yes	36	11.6%	
		No	273	88.4%	
		Total	309	100%	
	Street trade	Yes	84	27.2%	
		No	225	72.8%	
		Total	309	100%	
	Car washing and protection	Yes	85	27.3%	
		No	224	72.7%	
		Total	309	100%	
Income	≤ 30 ETB		142	45.5%	
	31–50 ETB		122	39.1%	
	≥51 ETB		48	15.4%	
	Total		312	100%	
Reasons to live on the street	Search of job	Yes	148	47.4%	
		No	164	52.6%	
		Total	312	100%	
	Peer pressure	Yes	90	28.8%	
		No	222	71.2%	
		Total	312	100%	
	Death of parents	Yes	76	24.4%	
		No	236	75.6%	
		Total	312	100%	
	Home violence	Yes	72	23.1%	
		No	240	76.9%	
		Total	312	100%	

More than a two third of the children had once been in school and nearly half of all the children were in the first cycle of primary education. Currently only 19(6.1%) are following their formal education. Search of job 148(47.4%), peer pressure 90(28.8%) and death of parents 76(24.4%) were the commonest factors that caused the children to join street life. Almost all studied street children are involved in at least one income generating activity on the street 309(99%). Among these activities are carrying goods 226(72.4%), street vending 84(27.2%) and car washing and protection 85(27.3%). The median daily income of the children was 35 ETB with IQR of 20 ETB(30-50ETB) (Table 1).

**Fig. 2:**
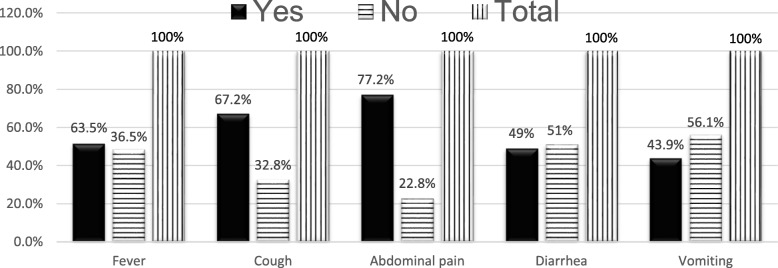
Common symptoms of illnesses for studied street children in Jimma town,2019

Half of studied children had lost one or both or don’t know whereabouts of their biological parents. Among those whom their biological parents died, 34(26.0%)lost both their father and mother. In addition, far more than half respondents198(63.5%) had history of recent illness with the commonest being in the last 2 weeks 95(30.4%). Fever, abdominal pain and vomiting were the most common symptoms of their recent illness (Fig. 2). Almost half of all study participants 149(47.8%) sustained varying levels of injury in 1 month preceding the study. The commonest source of injuries was fighting, falling, car accident and being beaten by police. Only half of the children who has been ill or injured received treatment121(51.5%). Shortage of money to pay for treatment was the reason for more than half not to seek treatment 61(53.5%).

### Prevalence of intestinal parasitic infections

Intestinal parasites were detected from almost two third of the stools of study participants 208(66.7%);95% CI (61.2–72.1). Polyparasitism (detection of two or more parasitic species from stool specimen) was observed in 33(15.9%) participants with positive stool samples, and all were dual polyparatism. A total of nine parasitic species were detected from stool specimens. The parasites fall under categories of soil transmitted helminths, *S. mansoni*, cestodes and protozoans. The category of STH and *S. mansoni* contributed to 95.2% of detected parasitic species. The commonest detected species was *A. lumbricoides* 121(58.2%) and the rarest was pathogenic amoebic species (*Entameoba histolytica*) 7(3.4%) (Table 2).

**Table 2:** Intestinal parasitic species recovered from stools of studied children of the street in Jimma town, 2019

Species of parasites	Frequencies	Percentages	Remark
*A. lumbricoides*^*a*^	121	58.2%	
*T. trichuria*^*a*^	28	13.5%	
*S. mansoni*	24	11.5%	
*Taenia species*	11	5.3%	
Hookworms^*a*^	16	7.7%	
*Hymenolepis nana*	9	4.3%	
*Strongloides stercolaris*	2	1%	
*Giardia intestinalis*	22	10.6%	
*E. histolytica*	7	3.4%	

^a^*STH* Soil Transmitted Helminths

### Factors associated with intestinal parasitic infections among street children

Bivariable and multivariable logistic regression was used to assess factors associated with IPIs among street children in Jimma town. Majority of independent variables showed significant association on the Bivariable model (Table 3). After taking the independent variables to the multivariable model, IPI status was found to be significantly associated with status of fingernails at the time of data collection, shoe wearing at the time of data collection, swimming practice, practice of eating street food and knowledge of at least one route of transmission of IPIs (Table 3).

**Table 3:** Bivariable and multivariable logistic regression results of factors associated with intestinal parasitic infections among street children in Jimma town,2019

Independent variable categories		IPI status		COR (95%CI for COR)	*P*-Value	AOR (95%CI for AOR)	*P*-Value*
		Positive (%)	Negative (%)				
Sex	Male	187(60%)	97(31%)	1			
	Female	21(6.7%)	7(2.3%)	1.5(0.64–3.8)	0.33		
Age	12–14	142(45.5%)	47(15%)	2.6 (1.6–4.23)	< 0.001		
	15–18	66(21.2%)	57(18.3%)	1			
Income	<35ETB	118(37.9%)	46(14.7%)	1.65(1.02–2.65)	0.038		
	> = 35ETB	90(28.8%)	58(18.6%)	1			
Educational status	Never joined	78(25%)	24(7.7%)	2.33(1.15–4.7)	0.018		
	Grade 1–4	98(31.4%)	57(18.3%)	1.23(0.66–2.3)	0.50		
	Grade 5–8	32(10.2%)	23(7.4%)	1			
Toilet use	Yes	97(31%)	71(22.8%)	1			
	No	111(35.6%)	33(10.6%)	2.46 (1.5–4.04)	< 0.001		
Hand washing after toilet	Yes	84(26.9%)	68(21.8%)	1			
	No	124(39.7%)	36(11.6%)	2.8 (1.7–4.5)	< 0.001		
Wash fruits and vegs. Before eating	Yes	52(16.7%)	47(15.1%)	1			
	No	156(50%)	57(18.3%)	2.5 (1.5–4.07)	< 0.001		
Cleanliness of fingernails	Clean	55(17.7%)	54(17.3%)	1			
	Not clean	153(49%)	50(16%)	3.0 (1.8–4.9)	< 0.001		
Status of finger nails	Trimmed	62(19.9%)	62(19.9%)	1			
	Not trimmed	146(46.7%)	42(13.5%)	3.5 (2.1–5.7)	< 0.001	2.03(1.02–4.0)*	0.045*
Nail trimming method	Tooth	125(40%)	37(11.9%)	2.72 (1.7–4.4)	< 0.001		
	Blade/others	83(26.6%)	67(21.5%)	1			
Hand washing before and after meals	Yes	96(30.7%)	76(24.4%)	1			
	No	112(35.9%)	28(9%)	3.2 (1.9–5.3)	< 0.001		
Having shoes	Yes	122(39.1%)	82(26.3%)	1			
	No	86(27.6%)	22(7%)	2.6 (1.5–4.5)	0.001		
Shoe wearing frequency	Regularly	64(31.4)	64(31.4%)	1			
	Irregularly	58(28.4%)	18(8.8%)	3.2 (1.7–6.0)	< 0.001		
Current shoe wearing	Yes	63(30.9%)	68(33.3%)	1			
	No	59(28.9%)	14(6.9%)	4.5 (2.3–8.9)	< 0.001	3.8(1.78–8.2)*	0.001*
Practice of Swimming	Yes	161(51.6%)	60(19.2%)	2.5 (1.5–4.2)	< 0.001	2.5(1.24–5.0)*	0.011*
	No	47(15%)	44(14.1%)	1			
Eating street foods	Yes	185(59.3%)	77(24.7%)	2.8 (1.52–5.2)	0.01	2.2(1.04–5.0)*	0.049*
	No	23(7.4%)	27(8.6%)	1			
Eating leftover foods	Yes	181(58%)	71(22.8%)	3.1 (1.74–5.5)	< 0.001		
	No	27(8.6%)	33(10.6%)	1			
Eating raw vegetables	Yes	147(47.1%)	56(17.9%)	2.0 (1.3–3.4)	0.004		
	No	61(19.5%)	48(15.4%)	1			
Source of drinking water	Protected	138(44.2%)	85(27.3%)	1			
	Unprotected	70(22.4%)	16(5.1%)	2.7(1.28–4.03)	0.005		
Knowledge of route of transmission of IPs	Yes	75(24%)	71(22.8%)	1			
	No	133(42.6%)	33(10.6%)	3.8(2.3–6.3)	< 0.001	2.5(1.25–5.0)*	0.01*

Status of finger nail at the time of data collection was found to be an independently associated factor with IPI status; AOR: 2.03, 95% CI (1.02–4.06). According to this finding, Street children whose finger nails are not trimmed were twice more likely to acquire IPIs when compared with children who had their finger nails trimmed. Shoe wearing at the time of data collection was also one of the significantly associated variables with IPIs; AOR = 3.8, 95% CI (1.8–8.2). The finding indicated that, children who were bare footed at the time of data collection were roughly four times more likely to acquire infections when compared with children who put on their shoes. Children who swim in unprotected water bodies were more than twice likely to acquire IPIs when compared with those who do not swim at all; AOR = 2.5, 95% CI (1.24–5.04).

In addition, eating street food was found to rise the likelihood of acquiring these infections and children who ate street food had more than twice odds of being infected when compared with those who don’t eat street food; AOR = 2.24 95% CI (1.004–5.02). Lastly, knowledge about the routes of transmission of these diseases was found to be significantly associated with the infection status: AOR 2.5, 95% CI (1.25–5.0). Accordingly, children who don’t know at least one route of transmission of IPIs were more than twice more likely to be infected when compared with those who know at least one route of transmission (Table 3).

## Discussion

In this study, the prevalence of intestinal parasitic infections among street children was found to be 66.7%;95% CI (61.2–72.1) and concurrent infection with two or more parasites was 15.9%. The predominant parasitic species were *A. lumbricoides*, *T. trichuria*, *S. mansoni* and hookworms. *G. intestinalis* was the commonest of protozoan species. The current prevalence of IPIs and STH in the town among this segment of population is categorized as high rate of infection demanding bi annual MDA administration according to the WHO. Several factors such as status of fingernails, practice of swimming in unprotected water bodies, shoe wearing, eating street food and knowledge of routes of transmission of IPIs were found to be associated with intestinal parasitic infections among street children. This indicates that street children are still at risk of developing morbidities from these diseases. Furthermore, these children may serve as a source of infection for the wider community and need to be considered in ongoing interventions by giving special emphasis on identified factors to meet the global and national targets of eliminating these diseases as a public health problem.

The current finding of the prevalence of IPIs in the study area is in line with a report from a study conducted on street dwellers in Addis Ababa which put the prevalence as 71.8% [19]. Similar finding was also reported from Sudan that stated the prevalence of IPIs among street children in Khartoum as 71.7% [20]. In addition, the finding from this study is comparable with a finding from Philippines among street children that put the prevalence of IPIs as 62% [21]. The composition of the species of parasites were also similar in that *A. lumbricoides, T. trichuria* and hookworms were the predominant parasites.

The current finding is lower than a report from a study conducted on street beggars in Jimma town which stated the prevalence as 89.7% [22]. The variation may be due to the difference in timing of the studies and the sample size. The study in Jimma among street beggars was conducted in 2010. In the year, MDA was not intensified since the national master plan on STH control was launched in 2013 by the Federal Ministry of Health in Ethiopia. As a result, the transmission of IPIs was thought to be extensive. Furthermore, the prior study used only wet mount method to assess the status of infection and this method is less sensitive supporting the above statement that the prevalence at the time could be even more than the stated. In addition, the study only involved 115 participants and may also be far from estimating the true population level prevalence among street dwellers.

The current study revealed that hygienic factors, feeding practices, leisure activities and knowledge of routes of transmission were associated with IPIs status (Table 3). Status of finger nails was found to be significantly associated with IPIs. This study revealed the increased likelihood of children with untrimmed finger nails to be infected with IPIs. This finding is supported by studies conducted on school aged children in Ambo and Butajira towns [23, 24]. The explanation would be possible presence of disease causing parasites underneath the nails with dirt [25]. The egg can be ingested with food to cause IPIs as most of studied children do not have practice of hand washing before and after meals. In the current study near a half, 140/312(44.8%) of the street children do not wash their hands before and after meals.

Eating street food is also associated with IPIs status among street children. Street children who consume street food are roughly twice more likely to be infected with IPIs when compared with those who do not consume (Table 3. This finding is supported by studies conducted on street residents in Addis Ababa and school aged children in Mizan town [19, 26]. The finding from the current and previous studies show that consumption of street foods poses increased chance of infection with IPIs. This might be due to unhygienic preparation, storage and vending of street foods that makes them contaminated with disease causing pathogens including intestinal parasites and bacteria.

Shoe wearing status was another significantly associated variable with IPIs. In this study, barefooted children were found to be nearly four times more likely to acquire IPIs. Studies from different parts of Ethiopia supported this finding. These studies found that children who were bare footed at the time of data collection had increased likelihood of acquiring IPIs [27]. In contrary, shoe wearing children had lowered odds of acquiring infections [28–32]. This may be due to increased susceptibility of bare footed children to skin penetrating intestinal parasites like hookworm and protective effects of shoe wearing on IPIs.

Practice of swimming was one of the significantly associated variables with parasitic infection (Table 3). According to the current study, children who reported swimming in unprotected water source are more than twice more likely to be infected with IPIs when compared with non-swimmers. This finding is strengthened by reports from several studies conducted in South Ethiopia like Arbaminch zuria woreda and lake Hawassa area; Northern and Central Ethiopia like Amhara regional state, Haike and some in South Eastern Ethiopia [27, 30, 32–34]. This can be explained by the established fact that frequent contact with unprotected waterbodies could result in an infection by skin penetrating infections like Schistosomiasis. In addition, swallowing unclean water could result in infection by disease causing pathogens including protozoans.

Knowledge of routes of disease transmission helps peoples to take precautionary measures to prevent infections and consequent sufferings. This truth is revealed in this study in that, children who do not know any route of transmission of IPIs are more than two times likely to acquire infections when compared with those who know at least one (Table 3). This finding is supported by a report from a study conducted in Addis Ababa among street residents [19]. In addition, community trials revealed that health education on core aspects of intestinal parasites including ways of transmission significantly reduced magnitude of parasitic infections [35, 36].

In this study, variables like educational status, age, sex and other WASH related characteristics were not independently associated with intestinal parasitic infections unlike studies conducted among non-street dwelling school age children in different part of Ethiopia. This may be due to the variation these between populations and little variation among the street children when it comes to these variables as they are more concentrated towards one of the other categories.

### Limitations of the study

The limitation of this study was that the IPI statuses of the children was ascertained by a single stool specimen which could underestimate the prevalence of the measure. In addition, the study didn’t include intensity of infections for STH and *Schistosoma mansoni* infections.

## Conclusions

This study has found high prevalence of intestinal parasitic infections among street children in the study area. The parasites included soil transmitted helminths, *Schistosoma mansoni* and protozoa species. Several factors were found to be associated with infection status. These factors include shoe wearing, swimming habit, habit of eating street food, nail trimming status and knowledge of ways of transmission of intestinal parasites. Measures has to be taken to curb the problem by including them in mass drug administration and targeted health education towards identified factors.

## Data Availability

All data for this research article is available and can be accessed from the corresponding author at any time.

## Abbreviations

BSc: Bachelor of Science
CI: Confidence Interval
ETB: Ethiopian Birr
FIDO: Fayya Integrated Development Organization
HIV: Human Immune Virus
IPI: Intestinal Parasitic Infections
IQR: Inter Quartile Range
MDA: Mass Drug Administration
PSAC: Preschool Age Children
SAC: School Age Children
SDG: Sustainable Development Goals
SEA: South East Asia
SPSS: Statistical Package for Social Sciences
SSA: Sub Saharan Africa
STH: Soil Transmitted Helminths
UN: United Nations
UNICEF: United Nations International Children’s Fund
WASH: Water, Sanitation and Hygiene
WHO: World Health Organization
